# Multi‐Omics Profiling and Experimental Verification of Lysosomes‐Related Genes in Hepatocellular Carcinoma

**DOI:** 10.1111/jcmm.70225

**Published:** 2024-12-18

**Authors:** Zhiyong Wang, Guoliang Wang, Peng Zhao, Ping Sun

**Affiliations:** ^1^ Department of Gastrointestinal Surgery, Union Hospital, Tongji Medical College Huazhong University of Science and Technology Wuhan China; ^2^ Department of Hepatobiliary Surgery, Union Hospital, Tongji Medical College Huazhong University of Science and Technology Wuhan China

**Keywords:** CTSV, hepatocellular carcinoma, immunotherapy efficacy, lysosomes, molecular docking, prognosis

## Abstract

Lysosomes play a crucial role in regulating the growth, invasion and metastasis of different tumour types. However, the specific function of lysosomes in hepatocellular carcinoma (HCC) remains uncertain. We retrieved gene expression and clinical data from the TCGA and GEO databases for HCC samples and established a new lysosome‐associated prognostic therapeutic index (LAPTI) based on lysosome‐related genes through machine learning. We then systematically analysed clinical characteristics, functional enrichment, tumour immune microenvironment, molecular docking, chemotherapy response and immunotherapy response in HCC. LAPTI, composed of four lysosome‐related genes (CTSV, LAPTM4B, DNAJC6, AP1M2), is a reliable prognostic indicator for hepatocellular carcinoma patients and is validated in external data sets. Compared with the low LAPTI group, the high LAPTI group showed poorer prognosis and higher immune cell infiltration levels. We also observed that knocking down CTSV in vitro inhibited the proliferation and migration of hepatocellular carcinoma. This study provides valuable insights into the future clinical treatment of hepatocellular carcinoma by accurately assessing the prognosis of patients with hepatocellular carcinoma.

**Abbreviations**
ACC: Adrenocortical carcinoma
BLCA: Bladder Urothelial Carcinoma
BRCA: Breast invasive carcinoma
CESC: Cervical squamous cell carcinoma and endocervical adenocarcinoma
CHOL: Cholangiocarcinoma
COAD: Colon adenocarcinoma
CTLA4: cytotoxic T‐lymphocyte‐associated protein 4
DLBC: Lymphoid Neoplasm Diffuse Large B‐cell Lymphoma
DSS: Disease‐free survival
ESCA: Oesophageal carcinoma
GBM: Glioblastoma multiforme
GDSC: genomics of xrug Sensitivity in Cancer
GEO: Gene Expression Omnibus
GO: Gene Ontology
GSEA: gene set enrichment analysis
HNSC: Head and Neck squamous cell carcinoma
HR: hazard ratio
IC50: half maximal inhibitory concentration
ICB: immune checkpoint blockade
KEGG: Kyoto Encyclopedia of Genes and Genomes
KICH: Kidney Chromophobe
KIRC: Kidney renal clear cell carcinoma
KIRP: Kidney renal papillary cell carcinoma
KM: Kaplan–Meier
LAML: Acute Myeloid Leukaemia
LGG: Brain Lower Grade Glioma
LIHC: Liver hepatocellular carcinoma
LUAD: Lung adenocarcinoma
LUSC: Lung squamous cell carcinoma
MESO: Mesothelioma
NES: normalised enrichment score
OS: overall survival
OV: Ovarian serous cystadenocarcinoma
PAAD: Pancreatic adenocarcinoma
PCPG: Pheochromocytoma and Paraganglioma
PD‐1: programmed cell death 1
PD‐L1: programmed cell death 1‐ligand 1
PFS: Progression‐free survival
PPI: protein–protein interaction
PRAD: Prostate adenocarcinoma
READ: Rectum adenocarcinoma
ROC: receiver operating characteristic
SARC: Sarcoma
SKCM: Skin Cutaneous Melanoma
ssGSEA: single‐sample gene set enrichment analysis
STAD: Stomach adenocarcinoma
TCGA: The Cancer Genome Atlas
TGCT: Testicular Germ Cell Tumours
THCA: Thyroid carcinoma
THYM: Thymoma
TIDE: tumour immune dysfunction and exclusion
TMB: tumour mutation burden
UCEC: Uterine Corpus Endometrial Carcinoma
UCS: Uterine Carcinosarcoma
UVM: Uveal Melanoma

## 1 Background

Liver cancer is among the most frequent malignant tumours seen in clinical practice. According to data, there were 905,000 liver cancer diagnoses and 830,000 fatalities globally in 2020, with the number of new cases and deaths anticipated to rise by more than 55% by 2024 [1]. Hepatocellular carcinoma (HCC) is the most prevalent primary liver cancer. HCC's gradual start and absence of substantial early clinical signs have historically made it difficult to diagnose and treat [2]. As bioinformatics technology advances, more biomarkers are found and utilised for tumour detection, monitoring and therapy [3, 4]. As a result, using bioinformatics technology to investigate molecular biomarkers that are significantly associated with the diagnosis, prognosis and treatment response of liver cancer can help improve the efficiency of liver cancer diagnosis and treatment while also realising the potential clinical benefits of biomarker‐guided therapy [5, 6].

Lysosomes are subcellular organelles that serve an essential function in cellular homeostasis [7]. Lysosomal membrane permeabilization is a sign of lysosomal membrane instability, which causes lysosomal malfunction and is very harmful, culminating in inflammation or cell death [8]. Given lysosomes' critical involvement in a range of cellular processes, lysosomal malfunction is the cause of many human disorders.

In this study, we used machine learning and multiple statistical methods to create a lysosome‐associated prognostic therapeutic index (LAPTI) based on lysosome‐related genes. We identified four lysosome‐related genes (CTSV, LAPTM4B, DNAJC6 and AP1M2) that were significantly associated with the prognosis of HCC. The LAPTI was extensively validated for its ability to predict the prognosis in HCC. Furthermore, molecular docking technology was employed to discover possible small‐molecule medicines that bind specifically to CTSV's key target protein. Finally, we demonstrated in vitro that knocking down the CTSV gene dramatically reduced the proliferation and migration of hepatocellular carcinoma.

## 2 Materials and Methods

### 2.1 Data Sources

We obtained the expression data and clinical information for patients with hepatocellular carcinoma from the publically accessible TCGA (https://portal.gdc.cancer.gov/) and GEO (https://cancergenome.nih.gov/) databases (TCGA‐LIHC, G SE45267 and GSE121248). The TCGA‐LIHC data set was utilised as the training cohort, whereas the GSE45267 and GSE121248 combined cohorts from the GEO database were used as the external validation cohorts.

### 2.2 Lysosome‐Associated Gene Set

Lysosome‐related genes were discovered and extracted from the MsigDB database (https://www.gsea‐msigdb.org/gsea/msigdb). Table S1 shows the particular gene set.

### 2.3 Construction of Lysosome‐Related Prognostic Therapeutic Index (LAPTI)

The training set was TCGA‐LIHC, and univariate Cox regression was utilised to identify differentially expressed genes (logFC > 1.5 and adj *p*‐value < 0.05). Lysosome genes linked to prognosis were then discovered. The lysosome‐associated prognostic therapeutic index (LAPTI) was developed using the LASSO and multifactor Cox regression analyses. The lysosome‐associated prognostic therapeutic index (LAPTI) is calculated as LAPTI = Σ (ExP × Coef). Coef is the gene coefficient, and ExP is the gene expression level.

### 2.4 Validation and Evaluation of LAPTI

HCC patients were classified into high and low LAPTI groups based on the median LAPTI score. The Kaplan–Meier survival curve was used to compare the overall survival (OS) times of the two groups, and the receiver operating characteristic (ROC) curve was used to assess predictive capacity at 1 year, 3 years and 5 years. Multivariate and univariate Cox regression analyses were used to determine if LAPTI is a prognostic predictor independent of other clinical factors (age, gender, grade, stage, T stage, N stage and M stage). A nomogram was created using all independent prognostic indicators to predict 1‐year, 3‐year and 5‐year survival in HCC patients, and calibration curves were utilised to evaluate the prediction's accuracy.

### 2.5 Enrichment Analysis

To obtain a better understanding of the related genes' biological activities and pathways, we used the R package ‘Clusterprofiler’ to conduct GO and KEGG enrichment analysis on the DEGs between the clusters. In addition, gene set enrichment analysis (GSEA) was used in the molecular signature data sets (https://www.gsea‐msigdb.org/gsea/msigdb) to discover enriched pathways in the high LAPTI group.

### 2.6 Immune Infiltration Analysis

Immune cell infiltration abundance and immune‐related functional activity were compared between high and low LAPTI groups using TIMER, CIBERSORT, CIBERSORT‐ABS, MCpCOUNTER, QUANTISEQ, XCELL, EpIC algorithms and single‐sample gene set enrichment (ssGSEA) analysis, which were displayed as heatmaps and boxplots. The ESTIMATE analysis was used to calculate purity ratings for immune, stromal and tumour cells.

### 2.7 Immune Therapy Response and Drug Sensitivity Evaluation

The Tumour Immune Dysfunction and Exclusion (TIDE) instrument was used to predict immunotherapy response in HCC patients, and high TIDE scores were linked to poor immunotherapy outcomes. The pRRophetic R package was used to determine the half‐maximal inhibitory concentration (IC50) of chemotherapeutic and targeted medicines in HCC patients in order to study the relationship between LAPTI and chemotherapy drug sensitivity.

### 2.8 Molecular Docking

We utilised Schrödinger software to find small molecule medicines that attach to the target protein and ran molecular docking simulations. We obtained the protein structure of the target (CTSV‐7PK4) from the PDB database and natural small molecule medicines from the PubChem database (https://pubchem.ncbi.nlm.nih.gov/). The Glide module of the Schrödinger program was utilised to mimic CTSV's binding posture with the small molecule medication.

### 2.9 Cell Culture and Transfection

Human HCC cell lines (HepG2 and Huh7) were obtained from Procell Life Science Co. Cells were grown in DMEM media with 10% foetal bovine serum (FBS) and 5% CO_2_ at 37°C. The shRNA sequence for CTSV is provided in Table S2.

### 2.10 EDU

The EdU test was carried out using the YF 488 Click‐iT EdU Kit (UElandy, China). HepG2 and Huh7 cells were cultured in 96‐well plates after shRNA transfection. EdU labeling and Hoechst identification were done in accordance with the manufacturer's procedure. Finally, EdU pictures were acquired using a fluorescent microscope. The ratio of EdU‐positive cells was calculated as the proportion of blue to green fluorescent cells.

### 2.11 Wound Healing and Cell Migration Test

Transfected HepG2 and Huh7 cells were sown in six‐well plates and incubated at 37°C in a humidified incubator for 24 h. After reaching 70%–90% cell confluence, a 20 μL pipette tip was used to scrape the monolayer perpendicular to the bottom of the well. Unattached cells were then removed by washing twice with PBS. Images were collected at 0 and 24 h under a 4x microscope, and the location and breadth of the scratch were recorded. Transwell assays were utilised to determine cell invasion. Transwell assays were utilised to determine cell invasion. We utilised Transwell chambers with no matrigel covering. Transfected cells were introduced to the top chamber with 200 μL of serum‐free medium, whereas the bottom compartment received 800 μL of 10% FBS medium. A 24‐well plate was incubated at 37°C for 24 h. After cleaning the cells on top of the upper chamber with a cotton swab, the transplanted cells at the bottom of the membrane were fixed with 4% paraformaldehyde for 30 min before being stained with crystal violet for 30 min. Then, photos were taken under a microscope and tallied in three different fields.

### 2.12 Statistical Analysis

The statistical analysis in this research was carried out using R and GraphPad Prism. Independent sample t‐tests, Wilcoxon rank sum tests, one‐way ANOVA and Mann–Whitney U tests were used to determine group differences. Statistical significance was defined at *p* < 0.05, with significance levels of *p < 0.05, **p < 0.01, ***p < 0.001, ****p < 0.0001, and ns (not significant).

## 3 Results

### 3.1 Construction and Validation of LAPTI

**FIGURE 1:**
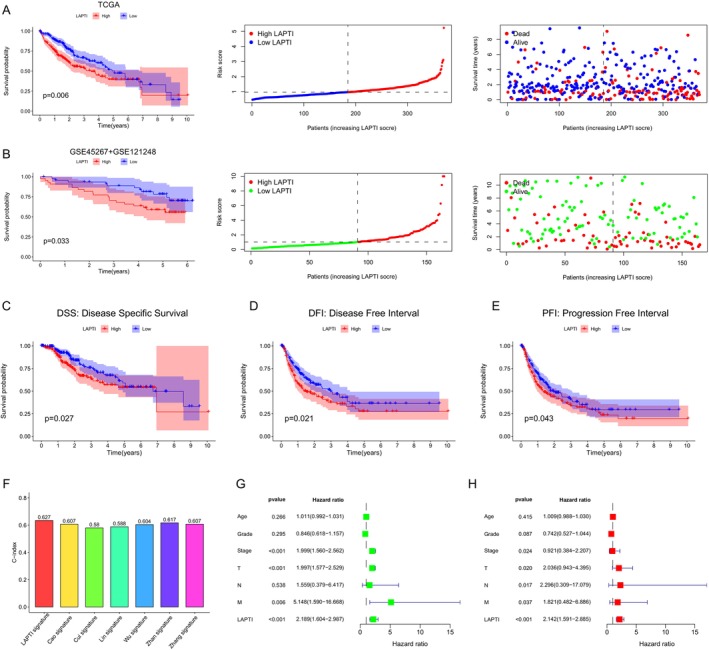
LAPTI predicts the prognosis of hepatocellular cancer patients. Kaplan–Meier survival curves of OS in the high and low LAPTI groups as well as rank‐point and scatter plots of LAPTI scores and patient survival status in the TCGA and GSE45267 + GSE121248 cohorts (A and B, respectively). (C–E) Kaplan–Meier survival curves for DSS, DFI and PFI in the TCGA cohort with high and low LAPTI levels. (F) Comparison of the LAPTI index to other BCa prognostic markers. (G–H) Univariate and multivariate regression analyses.

We discovered 191 lysosomal pathway‐related genes in the MsigDB database and included their clinical and pathological information in the gene expression profile in the TCGA‐LIHC training set. We performed univariate Cox regression analysis and multivariate Cox regression analysis to discover genes linked to prognosis. The collected prognostic genes were employed in LASSO regression analysis and multifactorial Cox analysis to determine the best prognostic group, and four genes (CTSV, DNAJC6, TREM2, and LAPTM4B) with independent prognostic values were eventually discovered. The expression levels and regression coefficients of these four genes were utilised to create the index LAPTI. LAPTI is determined by multiplying expCTSV by 0.0389, expDNAJC6 by 0.2119, expTREM2 by 0.0186 and expLAPTM4B by 0.0183. HCC patients were classified into high and low LAPTI groups based on their median LAPTI. The Kaplan–Meier analysis revealed that the overall survival (OS) of patients in the high LAPTI group was considerably lower than that of patients in the low LAPTI group (Figure 1A). GSE45267 + GSE121248, ICGC and GSE14520 were utilised as a validation cohort, and the results revealed that patients in the high LAPTI group had a poorer prognosis (Figures 1B and S1), which was similar to the TCGA findings. Subsequently, we discovered that patients with high LAPTI had a worse prognosis using Kaplan–Meier survival curves produced using several statistical techniques of patient survival time (Disease‐Specific Survival, Disease‐Free Interval, and Progression‐Free Interval) (Figure 1C–E). We also assessed the predictive efficacy of LAPTI in contrast to presently available models for predicting the prognosis of hepatocellular carcinoma. The findings revealed that LAPTI outperformed other models in predicting the prognosis of hepatocellular carcinoma patients (Figure 1F). Univariate and multivariate analyses were undertaken to establish if LAPTI is an independent prognostic indicator independent of other clinical parameters, and the findings revealed that LAPTI is an independent prognostic factor for HCC patients (Figure 1G,H).

**FIGURE 2:**
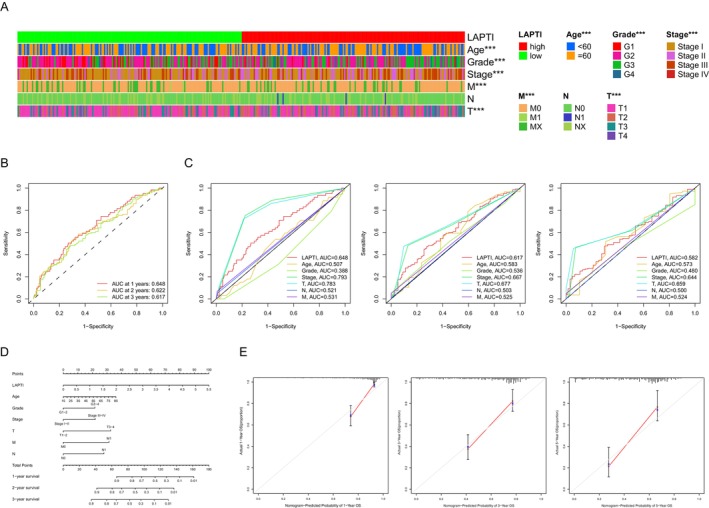
Correlation between LAPTI and clinical characteristics and construction of a nomogram. (A) Differences in LAPTI scores among clinical factors (age, grade, stage T, N and M). (B) ROC curves for LAPTI after 1, 3 and 5 years. (C) AUC comparison of LAPTI and other clinical parameters. (D) An LAPTI‐based nomogram for predicting the prognosis of hepatocellular carcinoma patients. (E) The nomogram's calibration curve.

We next examined the clinical and pathological features of the two LAPTI groups according to age, pathological grade, stage, T stage, N stage and M stage. The findings revealed substantial variations in clinical and pathological parameters between patients with low and high LAPTI, such as age, tumour grade, clinical stage, M stage and T stage (Figure 2A). We used a time‐dependent ROC analysis of LAPTI to assess the prognostic index's prediction efficacy for HCC patient survival. LAPTI had a higher area under the curve than clinical and pathological parameters such as pathological grade, age and N stage (Figure 2B,C). Based on the findings of the multivariable analysis, a nomogram was created to predict the OS of HCC patients using LAPTI, age, pathological grade, clinical stage and TNM stage. The anticipated 1‐year, 3‐year and 5‐year survival rates were congruent with clinical reality, and the calibration curve was quite accurate (Figure 2D,E).

### 3.2 GSEA Enrichment Analysis and LAPTI Correlation With the Immune Microenvironment

**FIGURE 3:**
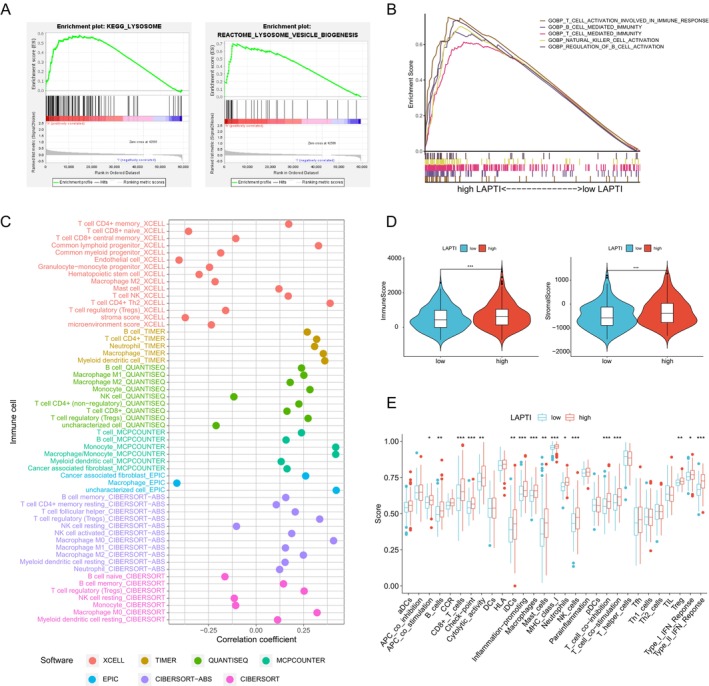
Tumour microenvironment assessment based on LAPTI. (A,B) GSEA study of patients with elevated LAPTI levels. (C) Correlation between LAPTI and immune cell infiltration using EPIC, XCELL, MCPCOUNTER, QUANTISEQ, CIBERSORT‐ABS, TIMER and CIBERSORT software. (D) Differences in immunological and stromal scores between high and low LAPTI groups. (E) The ssGSEA method is used to compare immune cells and immunological activities across groups with high and low LAPTI.

We conducted GSEA enrichment analysis on hepatocellular carcinoma patients based on LAPTI scores to identify enriched pathways related to LAPTI. The findings revealed that the high LAPTI group was considerably overrepresented in the lysosome and lysosome vesicle biogenesis signalling pathways (Figure 3A). The high LAPTI group was also more likely to have immune cell infiltration‐related signalling pathways, such as T‐cell activation implicated in immunological response, B‐cell‐mediated immunity, natural killer cell activation and regulation of B‐cell activation (Figure 3B). The relationship between LAPTI and immune cell infiltration was investigated using EPIC, XCELL, MCPCOUNTER, QUANTISEQ, CIBERSORT‐ABS, TIMER and CIBERSORT software. LAPTI was shown to be favourably linked with most immune cell infiltration levels (Figure 3C).

Furthermore, we investigated the relationship between LAPTI and immune cell infiltration levels, and the results revealed that patients in the high LAPTI group had higher immune and stromal scores than those in the low LAPTI group, implying that the high LAPTI group had a higher immune infiltration level than the low LAPTI group (Figure 3D). We also discovered that the high LAPTI group had more immune cell infiltration and immunological‐related activities than the low LAPTI group using the ssGSEA method (Figure 3E). These findings show that individuals with high LAPTI may have a high amount of immune infiltration in hepatocellular cancer.

### 3.3 Evaluation of the Efficacy of Immunotherapy

**FIGURE 4:**
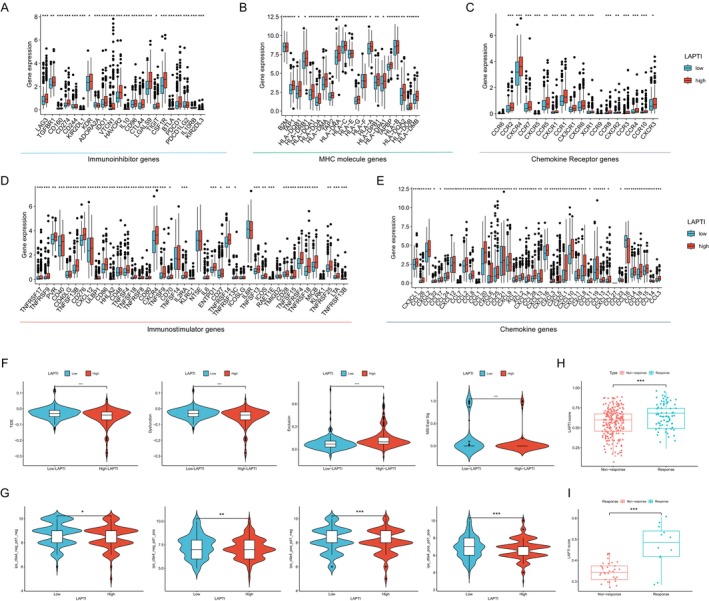
The efficacy of LAPTI in predicting the efficacy of immunotherapy. (A–E) Variations in the expression of common immunological checkpoints, cytokines and MHC molecules between high and low LAPTI groups. (F) Differences in TIDE and MSI scores between high and low LAPTI groups. (G) Differences in IPS scores between high and low LAPTI groups. LAPTI ratings differed across immunotherapy‐responsive and non‐responsive groups in the external immunotherapy validation data set.

We investigated the variations in the expression of immune checkpoint‐related genes between high and low LAPTI. The findings revealed that most immune checkpoint‐related genes were considerably overexpressed in high LAPTI (Figure 4A–E). In addition, we used the TIDE scoring method to measure HCC patients' immune escape potential and immunotherapy tolerance. The study found that exclusion and MSI were considerably greater in the high LAPTI group than in the low LAPTI group, but TIDE score and immunological dysfunction were significantly higher in the low LAPTI group than in the high LAPTI group. These findings indicate that individuals with high LAPTI may benefit more from immune checkpoint inhibitor medication (Figure 4F). By examining the relationship between the LAPTI group and the immune phenotype core (IPS), we discovered that the IPS score of the high LAPTI group was significantly higher than that of the low LAPTI group, implying that patients in the high LAPTI group may respond better to immunotherapy (Figure 4G). Finally, we gathered external real‐world immunotherapy data sets to evaluate the prognostic efficacy of LAPTI for immunotherapy response. The findings revealed that patients who reacted to immunotherapy had a considerably higher LAPTI score than those who did not respond (Figure 4H,I).

### 3.4 Pan‐Cancer Prognosis and Immunotherapy Efficacy

To further study the efficacy of LAPTI in predicting prognosis in patients with other malignancies, we utilised the LAPTI model formula to compute LAPTI for patients with other cancers and showed the survival curves of the high/low LAPTI groups and the TIDE score. Compared to the low LAPTI group, the high LAPTI group had a lower OS prognosis in BLCA, SARC, SKCM and UCEC but a better OS prognosis in CHOL and KIRC (Figure S2A). Patients with low LAPTI exhibited greater DSS survival in BLCA, SARC, SKCM and UCEC, but lower DSS survival in KIRC, compared to those with high LAPTI (Figure S2B). Patients with high LAPTI exhibited shorter DFI in COAD, KIRC and TGCY compared to those with low LAPTI (Figure S2C). In comparison to the high LAPTI group, the low LAPTI group showed longer PFI intervals in BLCA, TGCT, UCEC and UVM, but shorter PFI intervals in THYM (Figure S2D).

**FIGURE 5:**
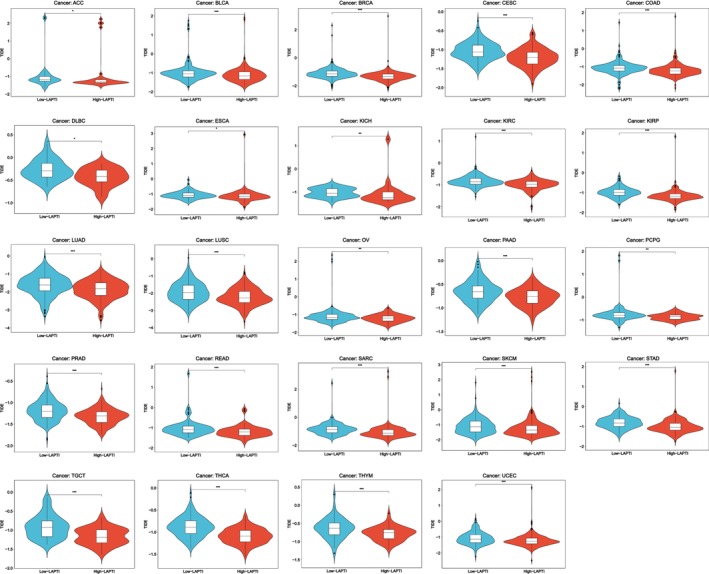
LAPTI score for evaluating immunotherapy efficacy in pan‐cancer.

Finally, we assessed the usefulness of the LAPTI score in predicting the success of immunotherapy in pan‐cancer patients. The results showed that the TIDE scores of the low/high LAPTI score groups were significantly different in ACC, BLCA, BRCA, CESC, COAD, DLBC, ESCA, KICH, KIRC, KIRP, LUAD, LUSC, OV, PAAD, PCPG, PRAD, READ, SARC, SKCM, STAD, TGCT, THCA, THYM and UCEC, indicating that in addition to BLCA, LAPTI score can also be used to evaluate the efficacy of immunotherapy in pan‐cancer and that the high LAPTI group has a better response to immunotherapy (Figure 5).

### 3.5 Drug Sensitivity Analysis

**FIGURE 6:**
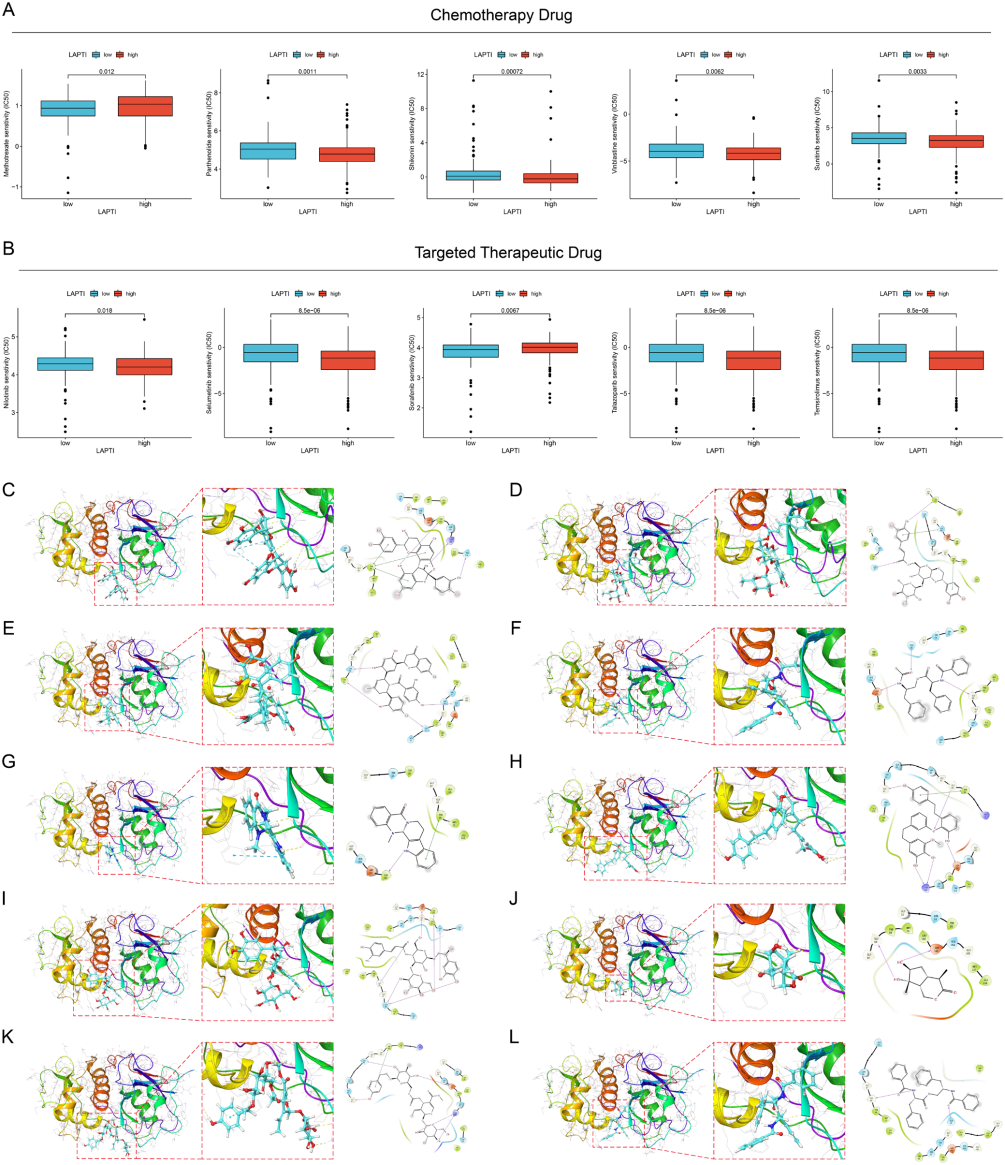
Value and molecular docking of LAPTI in predicting drug sensitivity. (A) Difference in IC50 values of common chemotherapeutic drugs between high/low LAPTI groups. (B) Difference in IC50 values of common targeted therapeutic drugs between high/low LAPTI groups. Molecular docking was used to screen candidate small molecules for target proteins. The figure shows the docking pose of the CTSV active pocket with Procyanidin A2 (C), Isocrenatoside (D), Guangsangon M (E), Patriscabratine (F), Isoevodiamine (G), Paleatin A (H), Hellicoside (I), Patriscabrol (J), Specneuzhenide (K) and Asperphenamate (L).

To test LAPTI's ability to forecast the sensitivity of chemotherapy medications, we looked for potentially effective anti‐liver cancer chemotherapy agents and targeted treatments and calculated their half‐maximum inhibitory concentration (IC50) values. We discovered that the sensitivity of several commonly used chemotherapeutic medicines and targeted treatments varied dramatically between high and low LAPTI. Methotrexate's IC50 value was substantially higher in the high LAPTI group than in the low LAPTI group, indicating that methotrexate may have a greater therapeutic impact in the low LAPTI group (Figure 6A). The IC50 value of nilotinib was considerably lower in the high LAPTI group than in the low LAPTI group, indicating that nilotinib may have a greater therapeutic impact in the high LAPTI group (Figure 6B).

### 3.6 Molecular Docking of the Target Protein CTSV

Molecular docking is a structure‐based computer approach used to screen molecules. We acquired the CTSV (7PK4) protein structure from the PDB database and used molecular docking with natural small molecule drugs. The top 10 small compounds with the best affinity for the CTSV protein binding pocket (Procyanidin A2, Isocrenatoside, Guangsangon M, Patriscabratine, Isoevodiamine, Paleatin A, Hellicoside, Patriscabrol, Specneuzhenide and Asperphenamate) (Figure 6C–L). For example, patriscabratine forms hydrogen bonds with CTSV amino acid residues Gln‐19, Gly‐68 and Asp‐163, with Gly‐68 and Asp‐163 functioning as acceptors and Gln‐19 as a donor. Procyanidin A2 forms hydrogen bonds with the CTSV amino acid residues Leu‐162, Lys‐160 and Thr‐214 as well as a Pi‐cation link with Phe‐36. Leu‐162, Lys‐160 and Thr‐214 serve as hydrogen bond acceptors.

### 3.7 Silencing CTSV Inhibits the Growth and Metastasis of HCC Cells

**FIGURE 7:**
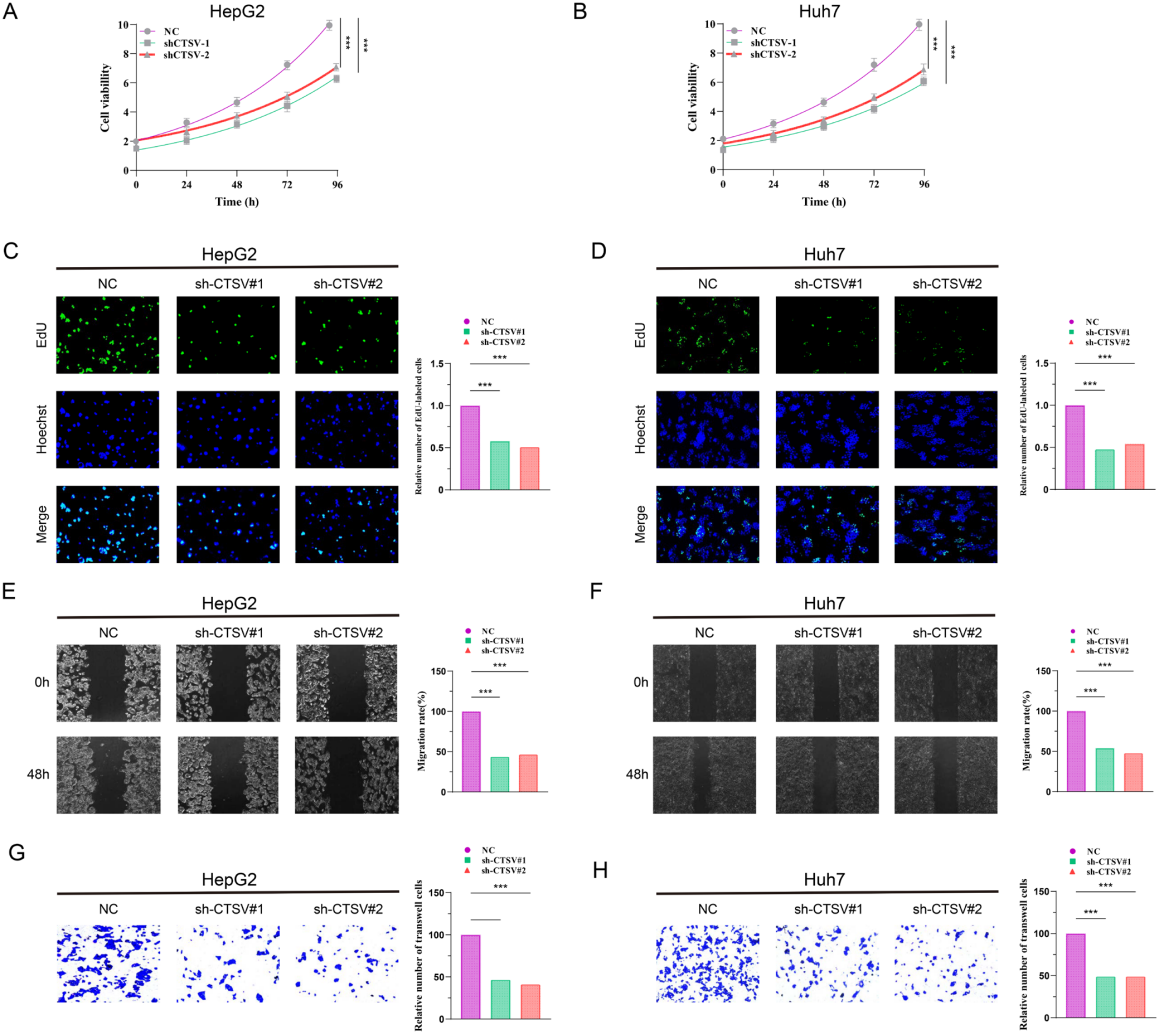
Knockdown of CTSV inhibits the proliferation and migration of HCC cell lines. (A, B) CCK8 assay shows that the number of HCC cells with CTSV knockdown is lower than that of sh‐NC cells at different time points. (C, D) EdU assay shows that the cell formation ability of CTSV knockdown cells is weaker than that of NC cells. (E–H) Cell scratch assay and Transwell assay observe the changes in the migration and invasion ability of CTSV knockdown cells compared with NC cells.

We confirmed LAPTI's predictive value in vitro. CTSV, one of the four LAPTI genes, is a lysosome‐associated gene implicated in the malignant development of certain malignancies (33298139). Therefore, we studied the biological role of CTSV in hepatocellular cancer. We transfected HepG2 and Huh7 cells with CTSV‐specific shRNA (sh‐CTSV) (Figure S3) and investigated its impact on HCC cell proliferation and invasion utilising EDU, CCK8, wound healing and transwell assays. CCK8 and EdU tests revealed that suppressing CTSV greatly decreased HCC cell growth (Figure 7A–D). Furthermore, inhibiting CTSV dramatically inhibited HCC cells' capacity to repair and migrate (Figure 7E–H). The findings imply that CTSV increases the growth and metastasis of HCC.

## 4 Discussion

Liver cancer is on the rise globally, and identifying novel treatment targets and molecular indicators associated with immunological prognosis would significantly increase the survival rate of liver cancer patients [9]. Lysosomes have a substantial impact on cancer progression and therapeutic responses [10]. This work developed a predictive and therapeutic assessment score for lysosomes based on four major lysosomal genes (CTSV, LAPTM4B, DNAJC6 and AP1M2), opening up new avenues for enhancing the efficiency and accuracy of HCC diagnosis and therapy. This research investigated the possible predictive usefulness of lysosome‐related genes in HCC using bioinformatics. We initially examined the expression of lysosome‐related genes in HCC tissues using the TCGA data set and identified 86 DEGs. Then, we utilised an unsupervised clustering technique to partition the HCC samples into Cluster A and Cluster B, yielding 46 inter‐cluster DE. Finally, utilising univariate Cox regression and LASSO regression analyses, four genes with independent prognostic significance were found, and the prognostic index LAPTI was effectively developed. We discovered that LAPTI may accurately predict the prognosis of individuals with hepatocellular cancer. We also discovered a natural small molecule medication that can target the lysosomal core target protein CTSV via molecular docking, and we confirmed in vitro that knocking down CTSV decreases the proliferation and invasion of hepatocellular carcinoma cell lines.

In this investigation, we discovered that patients with high LAPTI scores had a poor prognosis and that LAPTI may be utilised as a prognostic predictor for HCC regardless of other clinical parameters. In terms of prediction accuracy, the LAPTI score prediction findings were compatible with the clinical scenario, with good prediction efficiency. Lysosome‐related genes were found to be closely related to the signalling pathways of Lysosome and Lysosome vesicle biogenesis, T‐cell activation involved in immune response, B‐cell‐mediated immunity, T‐cell‐mediated immunity, Natural killer cell activation and B‐cell activation regulation. These findings indicate that lysosome‐related genes are tightly linked to tumour incidence and development, metabolism and immunological prognosis. We also discovered that LAPTI scores were favourably linked with numerous immune cells. Based on the positive association between high LAPTI scores and the expression of several immune checkpoints, we suggest that LAPTI may be utilised to choose appropriate immune checkpoint inhibitors for HCC patients. Based on the fact that high LAPTI patients had a lower TIDE score than low LAPTI patients, we believe that immune checkpoint inhibitor medication may benefit them more.

The lysosome‐associated prognostic therapeutic index (LAPTI) consists of four lysosome‐associated genes: CTSV, LAPTM4B, DNAJC6 and AP1M2. Although these genes have been demonstrated to influence a variety of biological processes in malignancies, their biological roles in hepatocellular carcinoma deserve additional investigation [3, 11, 12, 13, 14, 15]. Using CTSV as an example, we conducted in vitro tests to validate this. A series of cell proliferation and migration tests demonstrated that CTSV accelerated the growth and metastasis of HCC. However, our work only examined cell culture levels, and animal models are required to determine if these LAPTI genes control HCC growth in vivo. In addition, our research has several limitations. First, we collected our analytic data from public sources, which may have resulted in some case selection bias. The influencing factors of immune infiltration have many, such as tumour heterogeneity, tumour mutation compliance, microsatellite instability, tumour histological type, immune cell auto‐factors and different methods of immune cell infiltration analysis. Furthermore, although we acquired numerous external data sets to corroborate the study's conclusions, we still need to collect a significant number of clinical case data to confirm the veracity of the findings. Although LAPTI has evident predictive significance in HCC, its underlying mechanism remains unknown, and additional clinical study is needed. As a result, we want to investigate whether signal or pathway LAPTI genes play a role in HCC as well as to confirm LAPTI's profound clinical relevance using a large number of fresh clinical samples.

## 5 Conclusion

To summarise, we created LAPTI, a unique prognostic index based on lysosomal genes. It is an independent prognostic factor for HCC with high predictive accuracy. Furthermore, the immunological significance, drug sensitivity and biological function of LAPTI in the occurrence of HCC were investigated, revealing that it is useful for customised prognosis monitoring of HCC.

## Author Contributions

**Zhiyong Wang:** conceptualization (equal), data curation (equal), formal analysis (equal), investigation (equal), methodology (equal), resources (equal), software (equal), supervision (equal), validation (equal), visualization (equal), writing – original draft (equal). **Guoliang Wang:** data curation (equal), investigation (equal), resources (equal), software (equal), supervision (equal), validation (equal), visualization (equal), writing – original draft (equal). **Peng Zhao:** conceptualization (equal), data curation (equal), investigation (equal), project administration (equal), resources (equal), software (equal), visualization (equal), writing – original draft (equal). **Ping Sun:** conceptualization (equal), formal analysis (equal), funding acquisition (equal), investigation (equal), methodology (equal), project administration (equal), resources (equal), software (equal), supervision (equal), visualization (equal), writing – review and editing (equal).

## Ethics Statement

The authors have nothing to report.

## Consent

The authors have nothing to report.

## Conflicts of Interest

The authors declare no conflicts of interest.

## Supporting information


**Figure S1.** LAPTI predicts the prognosis of hepatocellular cancer patients in the ICGC and GSE14520 cohorts.

**Figure S2.** Predictive value of LAPTI in other cancers. Kaplan–Meier survival curves were used to compare the OS (A), DSS (B), DFI (C) and PFI (D) of patients with high/low LAPTI in pan‐cancer.

**Figure S3.** QPCR validation of CTSV mRNA expression levels in HepG2 and Huh7 cells after transfection with shRNA.

**Table S1.** List of lysosome‐related genes.

**Table S2.** Sequence of CTSV knockdown.

## Data Availability

All data utilised in this study are included in this article and all data supporting the findings of this study are available on reasonable request from the corresponding author.
