# Supplementary figures and images for: Multi‐Omics Profiling and Experimental Verification of Lysosomes‐Related Genes in Hepatocellular Carcinoma
Source: J Cell Mol Med. 2024 Dec 18;28(24):e70225. doi: 10.1111/jcmm.70225 (PMC11655306; doi:10.1111/jcmm.70225)

ICGC

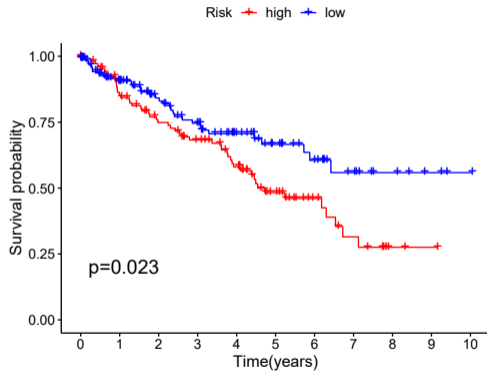

GSE14520

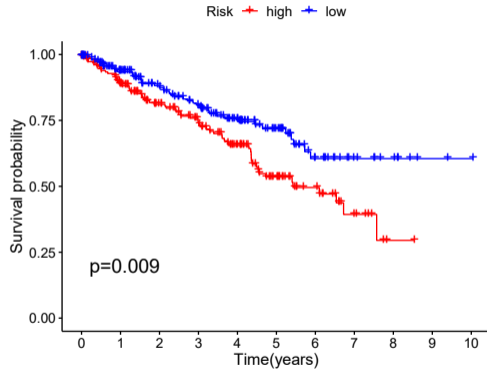

Supplement: Supplementary file 1 — Figure S1. LAPTI predicts the prognosis of hepatocellular cancer patients in the ICGC and GSE14520 cohorts. [file JCMM-28-e70225-s005.pdf]

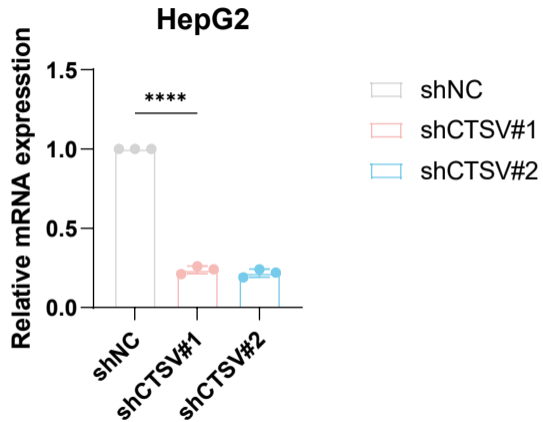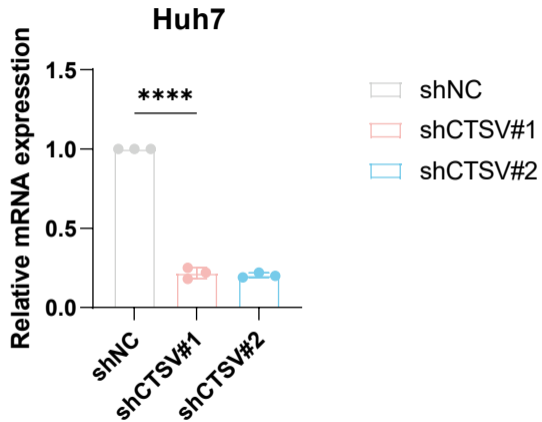

Supplement: Supplementary file 3 — Figure S3. QPCR validation of CTSV mRNA expression levels in HepG2 and Huh7 cells after transfection with shRNA. [file JCMM-28-e70225-s004.pdf]
