# Supplementary material for: Multi‐Omics Profiling and Experimental Verification of Lysosomes‐Related Genes in Hepatocellular Carcinoma
Source: J Cell Mol Med. 2024 Dec 18;28(24):e70225. doi: 10.1111/jcmm.70225 (PMC11655306; doi:10.1111/jcmm.70225)
Supplement: Supplementary file 2 — Figure S2. Predictive value of LAPTI in other cancers. Kaplan–Meier survival curves were used to compare the OS (A), DSS (B), DFI (C) and PFI (D) of patients with high/low LAPTI in pan‐cancer. [file JCMM-28-e70225-s002.pdf]

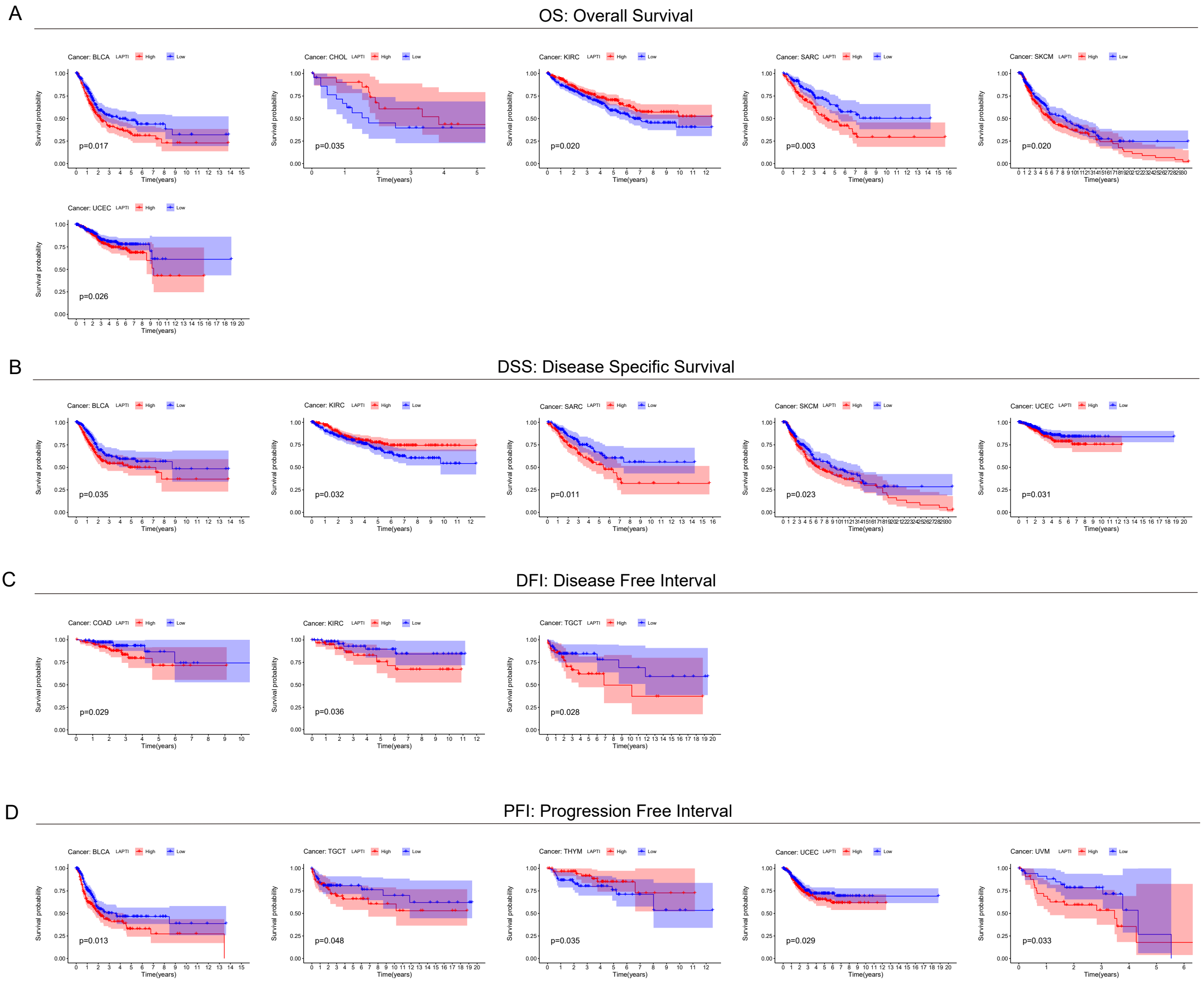
A
OS: Overall Survival
Cancer: BLCA
p=0.017
Cancer: CHOL
p=0.035
Cancer: KIRC
p=0.020
Cancer: SARC
p=0.003
Cancer: SKCM
p=0.020
Cancer: UCEC
p=0.026
B
DSS: Disease Specific Survival
Cancer: BLCA
p=0.035
Cancer: KIRC
p=0.032
Cancer: SARC
p=0.011
Cancer: SKCM
p=0.023
Cancer: UCEC
p=0.031
C
DFI: Disease Free Interval
Cancer: COAD
p=0.029
Cancer: KIRC
p=0.036
Cancer: TGCT
p=0.028
D
PFI: Progression Free Interval
Cancer: BLCA
p=0.013
Cancer: TGCT
p=0.048
Cancer: THYM
p=0.035
Cancer: UCEC
p=0.029
Cancer: UVM
p=0.033
LAPTI
High
Low
Survival probability
Time(years)
